# A 25-Hour Fast Among Quiescent Hereditary Coproporphyria and Variegate Porphyria Patients is Associated With a Low Risk of Complications

**DOI:** 10.5041/RMMJ.10490

**Published:** 2023-01-29

**Authors:** Yonatan Edel, Rivka Mamet, Iftach Sagy, Igor Snast, Ran Kaftory, Tomer Mimouni, Assi Levi

**Affiliations:** 1 Porphyria Center, Rabin Medical Center, Petah Tikva, Israel; 2 Medicine B, Assuta Ashdod Medical Center, Ashdod, Israel; 3 Ben-Gurion University of the Negev, Be’er-Sheva, Israel; 4 Sackler Faculty of Medicine, Tel Aviv University, Tel Aviv, Israel; 5 Rheumatology Unit, Soroka University Medical Center, Be’er-Sheva, Israel; 6 Dermatology, Rabin Medical Center, Petah Tikva, Israel

**Keywords:** Attack, emergency department, fast, porphyria, Yom Kippur

## Abstract

**Objective:**

In patients with acute hepatic porphyria (AHP), prolonged fasting is a known trigger of AHP attacks. Despite this, some Jewish AHP patients—mainly hereditary coproporphyria (HCP) and variegate porphyria (VP) patients—fast for 25 consecutive hours during the traditional Jewish holy day known as Yom Kippur. In this study, we evaluated the effect of the fast on these patients.

**Methods:**

A retrospective study and survey of AHP patients in Israel was carried out. Patients were asked whether they have fasted and whether any symptoms were induced by this fast. Patients’ medical records were reviewed for an emergency department (ED) visit following Yom Kippur between 2007 and 2019. Only 3 acute intermittent porphyria (AIP) patients reported fasting; they were excluded from analysis.

**Results:**

A total of 21 HCP patients and 40 VP patients completed the survey; 30 quiescent patients reported they fast, while 31 did not fast. The majority of fasting patients (96.67%) reported no symptoms following a fast. We found no statistically significant association between ED visits 1 week (0.26% in both fasting and non-fasting patients) or 1 month (2.1% visits in non-fasting versus 0.78% in fasting patients) following Yom Kippur. Of the symptomatic ED visits following a fast, none were defined as severe attacks.

**Conclusion:**

A 25-hour fast in stable HCP and VP patients did not increase the risk of an acute attack and can probably be regarded as safe.

## INTRODUCTION

The porphyrias are a group of rare metabolic disorders caused by either inherited or acquired abnormalities along the heme biosynthetic pathway.^1^,^2^ Each type of porphyria derives from a specific deficiency in one of the enzymes involved in the pathway and, accordingly, is characterized by a specific pattern of accumulation of heme precursors and typical clinical manifestations.^3^

Four types of porphyrias constitute the group of acute hepatic porphyrias (AHP): 5-aminolevulinic acid (5-ALA) dehydratase deficiency porphyria, acute intermittent porphyria (AIP), hereditary coproporphyria (HP), and variegate porphyria (VP). Acute hepatic porphyrias are characterized by acute attacks^4^ in which excessive heme is produced following an exposure to a trigger.^5^–^7^ The majority of acute attacks initially manifest as a combination of abdominal pain and mental symptoms such as severe fatigue and inability to concentrate, with or without autonomic dysfunction.^2^,^4^,^8^ Subsequently, abdominal pain may become worse, accompanied by nausea, vomiting, constipation, and signs of increased sympathetic activity, such as tachycardia and hypertension.^4^ Further exacerbation may follow with complete paralysis, respiratory failure, seizures,^9^ severe hyponatremia, and even death.^10^ This pattern of an acute attack is common to all types of AHP.

Many triggers of an AHP attack have been described, such as certain medications, alcohol consumption, infections, endogenous and exogenous hormones, and prolonged fasting.^2^ However, while prolonged fasting has been reported to trigger an attack in the setting of intercurrent illness and peri-surgical fasting, a crash diet, or bariatric surgery,^11^,^12^ it has not been thoroughly investigated in a controlled situation in quiescent disease.

Many people around the world practice fasting for various reasons: while some fast for religious reasons, others do so for philosophical ones or due to the belief fasting is healthy.

The Day of Atonement, also referred to as Yom Kippur, is the highest Jewish holy day. According to tradition, on Yom Kippur, a person’s destiny for the following year is supposedly “sealed.” Jewish people traditionally observe this holy day with an approximate 25-hour period of fasting. Hence, this study aimed to assess the impact of the Yom Kippur fast on AHP patients in a quiescent state.

## METHODS

A total of 111 patients whose diagnoses were recorded by the Israeli National Service for Biochemical Diagnoses of Porphyrias (INSP) between 1988 and 2019 were contacted by telephone and asked to participate in a survey. All cases were diagnosed according to known and well-established international criteria, as previously described.^6^ Patients were included if they were 16 years of age or older.

### Survey and Data Collection

This cross-sectional study was based on a telephone survey conducted between February 1, 2019, and March 1, 2020. Structured interviews were conducted by a single well-trained medical professional.

The survey collected demographic information including age, sex, comorbidity, and type of porphyria. Patients were asked about symptom type and aggravating factors such as alcohol consumption, fasting, smoking, and medications. The full questionnaire (translated into English) is provided in the supplementary material.

Next, the electronic medical records of all included patients were retrospectively reviewed for emergency department visits over a 13-year period (2007–2019). Each ED visit was counted separately.

We estimated that symptoms resulting from the Yom Kippur fast would start within one week of the triggering fast. To account for possible late effects of this fast we expanded the search for ED visits within 1 month of Yom Kippur.

A severe attack was defined as one which included at least one of the following: intensive care unit admission, severe hyponatremia (blood sodium level <125 mEq/L), or seizures.^13^,^14^

The study was approved by the institutional review board of Rabin Medical Center (RMC-35-19).

### Statistical analysis

Baseline characteristics and categorical variables were summarized using descriptive statistics and compared with the non-parametric test and exact Fisher’s *P* test as appropriate. The unit of analysis was the annual Yom Kippur fast per patient during the study period. The primary outcome was ED visit during the 7 days following Yom Kippur. Statistical significance was defined as *P*<0.05. The data were analyzed using WinPepi, version 11.65 for windows (Freeware, available at: http://www.brixtonhealth.com/pepi4windows.html).

## RESULTS

### Demographics and Baseline Characteristics

During 2019, we contacted 111 AHP patients, of whom 85 (77%) completed the survey: 40 with VP, 21 with HCP, and 24 with AIP. Of note, no patients had 5-ALA dehydratase deficiency porphyria. Since most of the AIP patients (21 out of 24) reported that they do not fast during Yom Kippur, this small group of AIP fasting patients was not included. Hence, the entire study group consisted of 61 AHP patients.

Patients had different mutations. None of our VP patients had the South African genotype since it is not as common in Israel. The main demographic characteristics of the HCP and VP survey participants are shown in Table 1. All patients were of Jewish ethnicity. The differences between fasting and non-fasting HCP and VP patients are shown in Table 2. There were no differences between the fasting and non-fasting groups regarding smoking and alcohol weekly intake (Table 2).

**Table 1:** Patients’ Main Demographic Characteristics.

Parameter	Value
Age (mean±SD)	46.5 (17.5)
Female (*n*, %)	30 (49.1%)
Variegate porphyria (*n*, %)	40 (65.6%)
Hereditary coproporphyria (*n*, %)	21 (34.4%)
Previously had porphyria symptoms (*n*, %)	40 (70.4%)
Previously visited ED due to porphyria (*n*, %)	23 (37.7%)
Previously received heme treatment (*n*, %)	5 (8.1%)
Total number of patient years for observed Yom Kippur fasts (2007–2019) (*n*, %)	383 (50%)

ED, emergency department.

**Table 2:** Differences Between Fasting and Non-fasting Hereditary Coproporphyria (HCP) and Variegate Porphyria (VP) Patients.

Parameter	Fasting Patients (*n*=30)	Non-fasting Patients (*n*=31)	*P* Value
Age (mean±SD)	50.7±17.5	43.6±17.1	0.7
Female (*n*, %)	15 (44.1%)	15 (48%)	0.89
Smokers (*n*, %)	10 (33%)	15 (48%)	0.23
Alcohol use >1 drink/week (*n*, %)	1 (3.3%)	3 (9.7%)	0.32
HCP patients, *n*=21 (*n*, %)	13 (57.5%)	8 (25.8%)	0.15
VP patients, *n*=40 (*n*, %)	17 (42.5%)	23 (74.2%)	
Previously had acute porphyria symptoms (*n*, %)	17 (56.6%)	26 (83.8%)	0.19
Previously visited ED due to porphyria (*n*, %)	10 (33%)	13 (41.9%)	0.48
Previously received heme treatment (*n*, %)	2 (6.6%)	3 (9.7%)	0.66
ED visits for porphyria symptoms within 1 week after fast (*n* visits/patient years)	1/383 (0.26%)	1/383 (0.26%)	1.0
ED visits for porphyria symptoms within 1 month after fast (*n* visits/patient years)	3/383 (0.78%)	8/383 (2.1%)	0.12

ED, emergency department.

A total of 43 of 61 (70.4%) patients stated they had previously experienced systemic symptoms (HCP 13/21; VP 30/40). Twenty-three (37.7%) patients stated they had previously visited the ED due to porphyria symptoms (5/21 HCP and 18/40 VP patients).

### Symptoms Due to Fasting

Information on symptoms and ED visits were collected from 61 patients, accounting for 793 patient years (Table 2). Twenty-seven patient years (3.4%) were excluded from the analysis since some of the participants were under the age of 16 years at the start of the study; hence, their data were analyzed only after they turned 16. This resulted in 31 patients (*n*=383 patient years) who did not fast on Yom Kippur and 30 (*n*=383 patient years) who did. All fasting and non-fasting patients reported doing so every year since their inclusion in the study. None of the patients who reported fasting during Yom Kippur had experienced an acute attack during the 2 years prior to their first fast. Of the 30 fasting patients, 29 (96.67%) patients reported no unusual symptoms following the fast, while 1 reported mild self-limiting symptoms. However, he continued fasting in the following years despite these symptoms.

No association was found between ED visits and fasting: within 1 week following Yom Kippur, ED visits due to porphyria symptoms occurred in 1/383 (0.26%) of both fasting and non-fasting groups (*P*=1). Neither was there an association between ED visits related to porphyria 1 month following the fast: 3/383 (0.78%) and 8/383 (2.1%) in the fasting and non-fasting groups, respectively (*P*=0.12).

Among fasting patients, as stated above, there were 3/383 (0.78%) ED visits during the 1 month following Yom Kippur versus 74/4323 (1.7%) visits during all other months in the study period (excluding the month following Yom Kippur) (averaging 5.69 visits per month during the study period) (*P*=0.5).

## DISCUSSION

An association between the lack of carbohydrate intake and the appearance of porphyria symptoms has long been described in both humans^11^,^12^ and rats.^15^,^16^ Conversely, high caloric intake and a higher insulin level were associated with lower biochemical disease activity.^17^ Furthermore, glucose treatment has an important role in the treatment of an acute attack.^2^ Nevertheless, while studies addressing extreme diets and carbohydrate deprivation are available, data regarding the association between controlled short-term fasts and porphyria symptoms are lacking.

In the current study, only 1 patient (3.3%) who fasted regularly on Yom Kippur reported having mild symptoms following the fast; he continued fasting despite these symptoms. No association was found between this controlled fast in stable AHP patients and ED visits; not even a single severe attack of porphyria was found during the month following Yom Kippur in the 383 fasts observed over 13 years. The absence of severe attacks could be partly attributed to the fact that attacks in previously diagnosed patients tend to be less severe than in previously undiagnosed patients, probably since other triggers are better controlled.^13^ Furthermore, although some of the fasting patients had experienced an acute attack in the past, they were all attack-free for at least 2 years prior to fasting.

This study has several limitations. First, due to its retrospective nature, it may be influenced by recall bias; however, patients’ reports were corroborated by the lack of difference in ED visits between the two groups. Second, since patients who previously experienced substantial symptoms tend to adhere more strictly to medical recommendations, including avoidance of fasting, as compared to quiescent patients, the possibility of a selection bias exists. Thus, the study results should only regard patients with quiescent disease and should not be used categorically in patients with active disease. Third, the low frequency of events may lead to a loss of statistical effect; however, since this is real-world data, we can assume that an attack due to fasting in this population is indeed rare. Finally, in this study we could not refer to AIP patients, since only 3 of 24 reported fasting during Yom Kippur. Since AIP patients tend to more commonly experience porphyria attacks (and some of them tend to chronically excrete high levels of urine porphobilinogen), we believe our results cannot be extrapolated to this population.

In conclusion, notwithstanding these limitations, this study is the first to show that a 25-hour fast among quiescent VP and HCP patients is associated with a low risk of complications and may be considered for specific patients. This finding should be the basis for further research.

## Supplementary Information



## Abbreviations

AHP: acute hepatic porphyria
AIP: acute intermittent porphyria
ED: emergency department
HCP: hereditary coproporphyria
VP: variegate porphyria
