# Supplementary material for: A 25-Hour Fast Among Quiescent Hereditary Coproporphyria and Variegate Porphyria Patients is Associated With a Low Risk of Complications
Source: Rambam Maimonides Med J. 2023 Jan 29;14(1):e0003. doi: 10.5041/RMMJ.10490 (PMC9888486; doi:10.5041/RMMJ.10490)
Supplement: Supplementary file 1 [file rmmj-14-1-e0003-am.docx]

This appendix has been provided by the authors for the benefit of readers

Supplement to: A 25-Hour Fast Among Quiescent Hereditary Coproporphyria and Variegate Porphyria Patients is Associated With a Low Risk of Complications

Edel Y, Mamet R, Sagi I, Snast I, Kaftory R, Mimouni T, Levi A. A 25-Hour Fast Among Quiescent Hereditary Coproporphyria and Variegate Porphyria Patients is Associated With a Low Risk of Complications. Rambam Maimonides Med J 2023;14 (1):e0003. doi:10.5041/RMMJ.10490

# The PORPHYRIA patient questionnaire

This two-part structured questionnaire comprises a first section covering demographic data, and a second section focusing on systemic features, including fasting habits. The questionnaire was conducted in Hebrew and was translated into English for purposes of presentation in this manuscript.

## Demographic Questionnaire

1. What is your ethnicity on your mother’s side and your father’s side?
2. What is your marital status? How many children do you have?
3. Are there any known porphyria patients or carriers in the family? If the answer is yes, what is the family relationship?
4. Try and estimate what percentage of your close family have been screened for porphyria.
5. Were you genetically screened for porphyria?
6. Do you suffer from any chronic illnesses? Could you elaborate?
7. Are you regularly taking medications for chronic conditions? Could you elaborate?
8. Females only: Do you take birth control pills or are you being treated with any other hormone therapy?
9. Do you smoke?
10. Are you aware that certain medications are forbidden for porphyria patients? If yes, how do you check if a medication is suited for you?
    1. Check with the prescribing doctor
    2. Check via the internet or lists I have received in the past
    3. Check with the Israeli National Service for the Biochemical Diagnoses of Porphyrias (INSP)
    4. Other
    5. I don’t check

## Neurocutaneous Porphyria – Systemic Manifestations Questionnaire:

1. Have you ever suffered from non-cutaneous disease manifestations attributed to porphyria?
2. Have you experienced abdominal pain induced by or related to porphyria [Yes/No]? If so, how often do you experience these symptoms [Daily/1–3 days per week/1–3 days per month/1–3 days per 3 months/1–3 days per year/<1 day per year]?
3. Could you elaborate on your abdominal symptoms (open question)?
4. Have you experienced vomiting induced by or related to porphyria [Yes/No]? If so, how often do you experience these symptoms [Daily/1–3 days per week/1–3 days per month/1–3 days per 3 months/1–3 days per year/<1 day per year]?
5. Could you elaborate on your vomiting symptoms (open question)?
6. Have you experienced limb pain induced by or related to porphyria [Yes/No]? If so, how often do you experience these symptoms [Daily/1–3 days per week/1–3 days per month/1–3 days per 3 months/1–3 days per year/<1 day per year]?
7. Could you elaborate on your limb pain (open question)?
8. Have you experienced limb numbness induced by or related to porphyria [Yes/No]? If so, how often do you experience these symptoms [Daily/1–3 days per week/1–3 days per month/1–3 days per 3 months/1–3 days per year/<1 day per year]?
9. Could you elaborate on your limb numbness (open question)?
10. Have you experienced muscle weakness induced by or related to porphyria [Yes/No]? If so, how often do you experience these symptoms [Daily/1–3 days per week/1–3 days per month/1–3 days per 3 months/1–3 days per year/<1 day per year]?
11. Could you elaborate on your muscle weakness (open question)?
12. Have you experienced anxiety episodes induced by or related to porphyria [Yes/No]? If so, how often do you experience these symptoms [Daily/1–3 days per week/1–3 days per month/1–3 days per 3 months/1–3 days per year/<1 day per year]?
13. Could you elaborate on your anxiety episodes (open question)?
14. Have you experienced confusion induced by or related to porphyria [Yes/No]? If so, how often do you experience these symptoms [Daily/1–3 days per week/1–3 days per month/1–3 days per 3 months/1–3 days per year/<1 day per year]?
15. Could you elaborate on the confusion you experienced (open question)?
16. Have you experienced seizures induced by or related to porphyria [Yes/No]? If so, how often do you experience these symptoms [Daily/1–3 days per week/1–3 days per month/1–3 days per 3 months/1–3 days per year/<1 day per year]?
17. Could you elaborate on your seizures (open question)?
18. Were you ever hospitalized or treated for any of the above mentioned symptoms? If so, try and estimate the number of times you have been hospitalized.
19. Were you ever treated intravenously for porphyria? If so, were you treated with glucose or heme?
20. Do you use painkillers? If so, try and estimate the frequency in which you use painkillers [Several times a day/Once a day/Several times a week/Several times a month/Less than several times a month].
21. Have any of your family members experienced one of the symptoms mentioned above? If so, what is his or her familial relationship?
22. Do you drink alcohol? If so, try and estimate the frequency of your drinking (one alcoholic drink = at least 1/3 of a beer, or 1/2 a glass of red wine, or 2/3 of a shot) [2 alcoholic drinks per day /1 alcoholic drink per day /Less than one alcoholic drink per day/Several alcoholic drinks per week].
23. If you do drink alcohol, do you experience any symptoms during or after drinking (a few days up to a week after you drink)? Do you experience any abdominal pain/limb pain/weakness/anxiety/ confusion/restlessness?
24. Do you smoke? If so, try and estimate the frequency of your smoking [20 cigarettes or more per day/10–20 cigarettes per day/1–10 cigarettes per day/less than 1 cigarette per day].
25. If you do smoke, do you experience any symptoms during or after smoking (a few days up to a week after you smoke)? Do you experience any abdominal pain/limb pain/weakness/anxiety/confu­sion/restlessness?
26. Do you fast for 25 hours on Yom Kippur day? At what age did you start fasting? Do you fast on any other events?
27. If you do fast, do you experience any symptoms during or after fasting (a few days up to a week after fasting)? Do you experience any abdominal pain/limb pain/weakness/anxiety/confusion/ restlessness?
28. Do you use or have ever used any drugs (such as cocaine, heroin, cannabis)?
29. If so, did you experience any symptoms during or after drug use (a few days up to a week after use)? Did you experience any abdominal pain/limb pain/weakness/anxiety/confusion/restlessness?
30. The following question is designated for women: Do you experience or have you experienced any unusual abdominal pain during the week before menstruation/third cycle week? Do you experience any limb pain/weakness/anxiety/confusion/restlessness?
